# Prognostic models for breast cancer: a systematic review

**DOI:** 10.1186/s12885-019-5442-6

**Published:** 2019-03-14

**Authors:** Minh Tung Phung, Sandar Tin Tin, J. Mark Elwood

**Affiliations:** Epidemiology and Biostatistics, School of Population Health, The University of Auckland, Private Bag 92019, Auckland, 1142 New Zealand

**Keywords:** Breast cancer, Prognostic model, Predictive model, Mortality, Survival, Recurrence, Prognosis, Nottingham prognostic index, Adjuvant!Online, PREDICT

## Abstract

**Background:**

Breast cancer is the most common cancer in women worldwide, with a great diversity in outcomes among individual patients. The ability to accurately predict a breast cancer outcome is important to patients, physicians, researchers, and policy makers. Many models have been developed and tested in different settings. We systematically reviewed the prognostic models developed and/or validated for patients with breast cancer.

**Methods:**

We conducted a systematic search in four electronic databases and some oncology websites, and a manual search in the bibliographies of the included studies. We identified original studies that were published prior to 1st January 2017, and presented the development and/or validation of models based mainly on clinico-pathological factors to predict mortality and/or recurrence in female breast cancer patients.

**Results:**

From the 96 articles selected from 4095 citations found, we identified 58 models, which predicted mortality (*n* = 28), recurrence (*n* = 23), or both (*n* = 7). The most frequently used predictors were nodal status (*n* = 49), tumour size (*n* = 42), tumour grade (*n* = 29), age at diagnosis (*n* = 24), and oestrogen receptor status (*n* = 21). Models were developed in Europe (*n* = 25), Asia (*n* = 13), North America (*n* = 12), and Australia (*n* = 1) between 1982 and 2016. Models were validated in the development cohorts (*n* = 43) and/or independent populations (*n* = 17), by comparing the predicted outcomes with the observed outcomes (*n* = 55) and/or with the outcomes estimated by other models (*n* = 32), or the outcomes estimated by individual prognostic factors (*n* = 8). The most commonly used methods were: Cox proportional hazards regression for model development (*n* = 32); the absolute differences between the predicted and observed outcomes (*n* = 30) for calibration; and C-index/AUC (*n* = 44) for discrimination.

Overall, the models performed well in the development cohorts but less accurately in some independent populations, particularly in patients with high risk and young and elderly patients. An exception is the Nottingham Prognostic Index, which retains its predicting ability in most independent populations.

**Conclusions:**

Many prognostic models have been developed for breast cancer, but only a few have been validated widely in different settings. Importantly, their performance was suboptimal in independent populations, particularly in patients with high risk and in young and elderly patients.

**Electronic supplementary material:**

The online version of this article (10.1186/s12885-019-5442-6) contains supplementary material, which is available to authorized users.

## Background

Breast cancer is the most common cancer in women worldwide [1]. The disease is highly heterogeneous with wide variations in prognosis [2]. Prognosis means the probability or risk that an outcome (such as deaths, complications, quality of life, pain, or disease regression) develops over a specific time, based on both clinical and non-clinical profiles [3]. In breast cancer patients, 5-year relapse-free survival (RFS) ranges from 65 to 80% [4], and 10-year overall survival (OS) ranges from 55 to 96% [5].

Prognosis for breast cancer is important in several ways. Firstly, it informs patients about the future course of their illness [3]. Two Australian surveys found that survival time information was desired by 87 and 85% of early and metastatic breast cancer patients, respectively [6, 7]. Secondly, prognosis is essential for breast cancer treatment: the more precise is the outcome predicted, the better a patient is allocated the right treatment [3, 8–10]. For example, patients whose prognosis is very poor may be considered for aggressive treatments, while those with a good prognosis may be saved from overtreatment and its related side-effects and financial costs [11, 12]. Thirdly, prognosis can be used for the inclusion and stratification of patients in experimental studies [8, 9]. Finally, prognosis helps policy makers compare mortality rates among hospitals and institutions [3, 13].

Many models have been developed to predict breast cancer prognosis. The number of models has increased rapidly, accompanying with the great variance in terms of patients included, methods of development, predictors, outcomes, presentations, and performance in different settings [11, 14]. Nevertheless, to our knowledge, only two reviews of prognostic models for breast cancer have been conducted, but with limitations. An earlier review reported 54 models that were developed between 1982 and 2001, with a focus on model development methods rather than model performance in different populations [11]. A more recent review included only 26 models published up to July 2012 [14]. This systematic review was undertaken to identify all prognostic models that have been published up to 2017, and to assess how the models performed in different settings.

## Methods

### Study search

A systematic search was conducted in EMBASE, PUBMED, Web of Science, COCHRANE, and in specific breast cancer and oncology websites, including: American Society of Clinical Oncology (ASCO) https://www.asco.org/, Journal of the National Comprehensive Cancer Network (JNCCN) http://www.jnccn.org/, Memorial Sloan Kettering Cancer Centre (MSKCC) https://www.mskcc.org/, MD Anderson Cancer Centre https://www.mdanderson.org/, Mayo Clinic http://www.mayoclinic.org/, and European Society for Medical Oncology (ESMO) http://www.esmo.org/. A manual search in the bibliographies of selected articles was also conducted. The search terms used were “prognostic model”, “breast cancer”, and their synonyms (see details in Additional file 1).

### Eligibility criteria

This review included all research articles that presented the development and/or validation of prognostic models for female breast cancer, were published in English prior to 1st January 2017 and were available in full text. The review was restricted to the models that were developed based on at least two different clinico-pathological factors and/or commonly used biomolecular factors, such as hormonal receptor status or human epidermal growth factor receptor 2 (HER2) status, and predicted mortality and/or recurrence of women who were diagnosed with primary breast cancer. Articles that reported the development of a model for specific patient groups (those with invasive ductal carcinoma or invasive lobular carcinoma, those who have undergone surgery) were included. Articles that presented the development of a model for rare histological subtypes of breast cancer or special types of patients (such as those with metastases, those with hormonal receptor negative or positive, those with node negative or positive, those with neoadjuvant or adjuvant therapy) were excluded due to their limited generalisability.

### Study selection and data extraction

Publications were screened in three levels - titles, abstracts, and full texts. From each selected article, relevant information was extracted into a data extraction sheet using the TRIPOD [15] and CHAMRS checklist [16], and included: authors, year of publication, objectives, name of models, study design, source of data, targeted populations, methods of development and/or validation, risk groups, outcomes, predictors, results of the development and/or validation, limitations and strengths.

The selected articles were categorised into three groups: those that presented model development, those that presented internal validation, and those that presented external validation. For the articles that presented the development of more than one model, we reviewed the best model only if the study indicated the best model, or we reviewed all the models presented if the study did not select the best model. Internal validation is defined here as the validation of a model in participants selected from the model development cohorts, or in patients recruited from the same source as in the development cohorts but at different times. External validation is defined as the validation of a model in patients from sources independent from the development cohorts [8].

### Assessment of risk of bias in individual studies

The risk of bias within individual studies was assessed by using a modified version of the QUIPS (QUality In Prognosis Studies) tool, which was originally designed to assess bias in studies of prognostic factors [17, 18]. The tool originally comprises six domains – Study Participation, Prognostic Factor Measurement, Outcome Measurement, Statistical Analysis and Reporting, Study Confounding, and Study Attrition, each of which is guided by three to seven prompting items. The last two domains were omitted as these are not relevant to the studies included in this review. The overall rating for each of the remaining four domains was assigned as low, moderate, or high risk of bias [17].

The risk of bias was assessed separately for development (and internal validation) studies and external validation studies. For articles that presented both model development and external validation, the risk of bias was assessed separately for each part. For articles that presented internal validation without model development, the risk of bias was assessed similarly to the external validation studies.

## Results

**Fig. 1:**
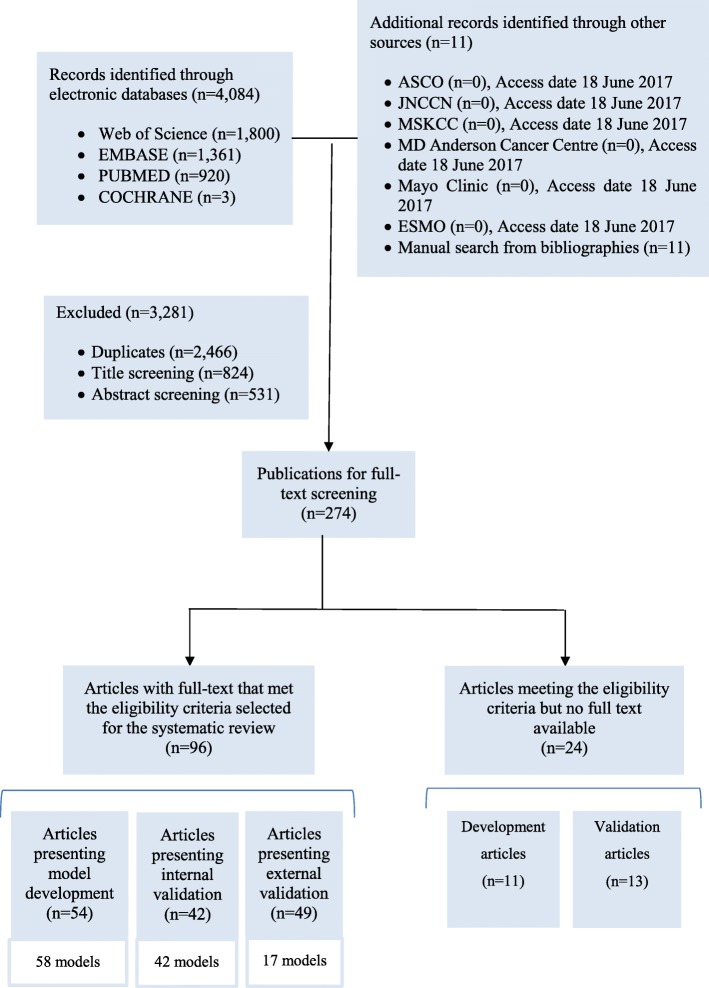
Flow diagram of the literature search process

The systematic search in the four databases generated 4084 records, supplemented by 11 publications found in other sources (Fig. 1**)**. We excluded 2466 duplicates. We screened the titles and then the abstracts of the remaining records and excluded 1355 records. We reviewed the full text of the remaining 274 articles and identified 96 eligible articles, of which 54 presented model development, 42 presented internal validation and 49 presented external validation. Twenty four studies that met the eligibility criteria but were not available in full text are presented in Additional file 2 (model development) and Additional file 3 (model validation).

### Study characteristics

The studies were published between 1982 and 2016, mostly retrospective and hospital-based. Participants were mostly from Europe, Asia, and North America (Table 1**)**.

**Table 1:** Characteristics of the studies selected for the systematic review

Characteristics	Model development studies	Internal validation studies	External validation studies
Number of studies	54 studies	42 studies	49 studies
Number of models	58 models	42 models	17 models
Year of publication	1982–2016	1982–2016	1987–2016
Study design			
Prospective	2 studies	2 studies	0 study
Retrospective	32 studies	23 studies	30 studies
Unknown	20 studies	18 studies	19 studies
Source of data			
Population-based	14 studies	11 studies	12 studies
Hospital-based	31 studies	29 studies	33 studies
RCT-based	6 studies	1 study	4 studies
Unknown	3 studies	2 studies	0 study
Sample size	75–433,272	30–433,272	48–387,262
Number of events			
Deaths	27–24,610	27–24,610	11–3902
Recurrences	5–1030	5–950	9–1188
Country of participants			
Europe	24 studies	22 studies	29 studies
North America	13 studies	8 studies	7 studies
Asia	11 studies	10 studies	11 studies
Others	2 studies (Australia)	0 study	3 studies (1 Australia. 1 New Zealand, 1 Brazil)
Strengths concluded by the authors of the selected studies			
Adhere to good practice	1 study	1 study	0 study
Large sample size	2 studies	2 studies	4 studies
Patients diagnosed recently	1 study	1 study	0 study
Homogeneous source of data	2 studies	2 studies	1 study
Low proportion of missing data	0 study	0 study	1 study
Weaknesses concluded by the authors of the selected studies			
Missing data	11 studies	11 studies	8 studies
Small sample size	3 studies	3 studies	9 studies
Patients treated with obsolete methods	4 studies	4 studies	3 studies
Heterogeneous source of data	3 studies	3 studies	0 study
Selection bias	2 studies	2 studies	0 study
Short-time follow-up	1 study	1 study	0 study

Of the 54 model development studies identified, 42 developed only one model, nine developed more than one model and selected the best performing model(s) [19–27], whereas three studies developed more than one model but did not select the best model(s) [28–30]. In total, we reviewed 58 models. More detailed information about each development study is presented in Additional file 4.

Among the 42 internal validation studies, 38 developed models and validated them, while four only validated the existing models: three studies validated the Nottingham Prognostic Index (NPI) [31–33], and one validated the Morphometric Prognostic Index (MPI) [34] (see details in Additional file 5).

Of the 49 external validation studies, 38 validated the existing models only, 10 developed new models and then validated them [19, 35–43], and one externally validated an existing model (Adjuvant!) and then developed a new model [44]. More detailed information about the external validation studies is presented in Additional file 6.

### Risk of bias in individual studies

The risk of bias was assessed for 54 studies in the development part (Table 2), and 53 studies in the validation part (Table 3). In all the four domains of the QUIPS tool, most studies had low or moderate risk of bias while only a small number were at high risk of bias.

**Table 2:** Risk of bias within model development studies

No	Citation	Study Participation	Prognostic Factor Measurement	Outcome Measurement	Statistical Analysis and Presentation
1	Asare et al. (2016) [107]	Low	Moderate	Moderate	Moderate
2	Baak et al. (1985) [108]	Low	Moderate	Low	Moderate
3	Broet et al. (1999) [109]	Low	Moderate	Moderate	Low
4	Brown et al. (1993) [110]	Moderate	Moderate	Low	Moderate
5	Bryan et al. (1986) [111]	Moderate	Low	Low	Low
6	Bucinski et al. (2005) [112]	High	Moderate	High	Moderate
7	Campbell et al. (2010) [19]	Low	Low	Moderate	Low
8	Chao et al. (2014) [20]	Moderate	High	Low	Moderate
9	Chen et al. (2016) [41]	Moderate	High	Low	Low
10	Cheng et al. (2006) [30]	Low	Moderate	High	Low
11	Choi et al. (2009) [25]	Low	High	Low	Moderate
12	Collan et al. (1994) [113]	Moderate	Low	Low	Moderate
13	de Laurentiis et al. (1999) [43]	Low	Moderate	Moderate	Moderate
14	Delen et al. (2005) [27]	Moderate	Moderate	Low	Moderate
15	Eskelinen et al. (1992) [10]	Moderate	Low	Moderate	Low
16	Fan et al. (2011) [56]	Low	Moderate	Low	Low
17	Fleming et al. (1999) [21]	Low	Moderate	Low	Moderate
18	Fuster et al. (1983) [114]	High	High	Low	Moderate
19	Gomez-Ruiz et al. (2004) [97]	Moderate	High	High	Moderate
20	Hawkins et al. (2002) [89]	Moderate	Low	Moderate	Moderate
21	Haybittle et al. (1982) [53]	Moderate	Low	Low	Moderate
22	Jerez Aragones et al. (2004) [115]	Moderate	High	Low	Moderate
23	Jerez et al. (2005) [26]	Low	Moderate	High	Low
24	Jhajharia et al. (2016) [116]	Moderate	High	Low	Moderate
25	M. Jung et al. (2013) [44]	Low	Moderate	Low	Moderate
26	Kim et al. (2012) [22]	Low	High	High	Low
27	Kim et al. (2016) [117]	Moderate	High	High	Low
28	Lisboa et al. (2003) [23]	Moderate	High	Low	High
29	Y.Q. Liu et al. (2009) [118]	Moderate	Moderate	Low	Moderate
30	Lovekin et al. (1991) [119]	High	Moderate	Low	Moderate
31	Masarwah et al. (2016) [55]	Moderate	Low	High	Low
32	Mazouni et al. (2011) [120]	Moderate	Moderate	Low	Low
33	Michaelson et al. (2011) [42]	Moderate	High	Low	Moderate
34	Musial et al. (2005) [121]	Moderate	Moderate	Low	Low
35	Ni et al. (2014) [122]	Moderate	Moderate	High	Low
36	Paik et al. (1990) [123]	Moderate	Low	Low	Moderate
37	Putter et al. (2006) [124]	Moderate	Moderate	Moderate	Moderate
38	Rakha et al. (2014) [90]	Moderate	Moderate	Low	Moderate
39	Ravdin et al. (2001) [125]	Low	High	Moderate	Moderate
40	Ripley et al. (1998) [29]	Moderate	High	High	Moderate
41	Sanghani et al. (2007) [126]	High	High	High	Moderate
42	Sanghani et al. (2010) [38]	Low	High	Moderate	Moderate
43	Shek & Godolphin (1988) [127]	Low	Moderate	Low	Moderate
44	Suen & Chow (2006) [91]	Low	Low	Moderate	Low
45	Tokatli et al. (2011) [28]	Moderate	Moderate	High	Low
46	Ture et al. (2009) [24]	Moderate	High	High	Low
47	van Belle et al. (2010b) [37]	Low	Low	High	Low
48	van Nes et al. (2010) [128]	Moderate	High	Moderate	Moderate
49	Wen et al. (2015) [129]	Low	Moderate	Low	Low
50	Wen et al. (2016) [57]	Low	Moderate	Low	Low
51	Wishart et al. (2010b) [40]	Low	High	Low	Low
52	Wishart et al. (2012) [35]	Moderate	Moderate	Low	Low
53	Wishart et al. (2014) [36]	Moderate	Low	Low	Low
54	Witteveen et al. (2015) [39]	Low	Moderate	High	Low

**Table 3:** Risk of bias within model validation studies

No	Citation	Study Participation	Prognostic Factor Measurement	Outcome Measurement	Statistical Analysis and Presentation
1	Aaltomaa et al. (1983) [130]	Low	Low	Low	Low
2	Albergaria et al. (2011) [75]	Moderate	Moderate	Low	Low
3	Alexander et al. (1987) [131]	Low	Low	Low	Low
4	Balslev et al. (1994) [48]	Low	High	Low	Low
5	Bhoo-Pathy et al. (2012) [61]	Low	Moderate	Moderate	Low
6	Campbell et al. (2009) [59]	Low	Moderate	Low	Low
7	Campbell et al. (2010) [19]	Moderate	Low	Moderate	Moderate
8	Carbone et al. (1999) [132]	Moderate	Low	Low	Low
9	Chen et al. (2016) [41]	Low	High	Low	Low
10	Chollet et al. (2003) [49]	Moderate	High	Low	Low
11	Collan et al. (1998) [98]	Moderate	Low	Low	Low
12	de Glas et al. (2014) [66]	Low	Moderate	Low	Low
13	de Glas et al. (2016) [69]	Low	Moderate	Low	Low
14	de Laurentiis et al. (1999) [43]	Moderate	Low	Moderate	Moderate
15	D’Eredita et al. (2001) [51]	Low	Low	Low	Low
16	Galea et al. (1992) [32]	Moderate	Moderate	Low	Moderate
17	Green et al. (2016) [133]	Low	Moderate	Low	Low
18	Hajage et al. (2011) [58]	Moderate	Low	Moderate	Low
19	Hearne et al. (2015) [47]	Low	Low	Low	Low
20	S.P. Jung et al. (2013) [134]	Low	Moderate	Low	Low
21	M. Jung et al. (2013) [44]	Low	Moderate	Moderate	Low
22	Kindts et al. (2016) [135]	Moderate	Moderate	Moderate	Low
23	Kollias et al. (1999) [31]	Moderate	Moderate	Low	Moderate
24	Kuo et al. (2012) [62]	Low	Low	Moderate	Low
25	Laas et al. (2015) [68]	Moderate	Moderate	Moderate	Low
26	Lende et al. (2010) [136]	Low	Moderate	Low	Low
27	M. Liu et al. (2010) [74]	Low	Moderate	Low	Low
28	Maishman et al. (2015) [137]	Moderate	Moderate	Low	Low
29	Megha et al. (2010) [70]	Moderate	Moderate	Low	Low
30	Miao et al. (2016) [138]	Moderate	High	Low	Low
31	Michaelson et al. (2011) [42]	Low	High	Low	Moderate
32	Mojir Sheibani et al. (2013) [65]	Low	Low	Moderate	Low
33	Mook et al. (2009) [64]	Low	Moderate	Moderate	Low
34	Okugawa et al. (2009) [52]	Low	Low	Low	Low
35	Olivotto et al. (2005) [45]	Low	High	Low	Low
36	Plakhins et al. (2013) [63]	Moderate	Moderate	Low	Low
37	Quintyne et al. (2013) [60]	Low	Moderate	Moderate	Low
38	Rejali et al. (2015) [54]	Moderate	Moderate	Moderate	Low
39	Ribelles et al. (1997) [139]	Moderate	Low	Low	Low
40	Sanghani et al. (2010) [38]	Moderate	Moderate	Moderate	Low
41	Sidoni et al. (2004) [71]	Moderate	Low	Low	Low
42	Sundquist et al. (1999) [72]	Low	Moderate	Low	Low
43	Todd et al. (1987) [33]	High	Low	Low	Moderate
44	van Belle et al. (2010a) [73]	Low	Moderate	Low	Low
45	van Belle et al. (2010b) [37]	Low	High	Low	Low
46	van Diest & Baak (1991) [34]	Low	Moderate	Low	Low
47	Wishart et al. (2010b) [40]	Low	High	Low	Low
48	Wishart et al. (2011) [46]	Low	High	Low	Low
49	Wishart et al. (2012) [35]	Low	High	Low	Low
50	Wishart et al. (2014) [36]	Low	Moderate	Low	Low
51	Witteveen et al. (2015) [39]	Low	Moderate	High	Low
52	Wong et al. (2015) [67]	Low	Moderate	Low	Low
53	Yadav et al. (2015) [50]	Moderate	Moderate	Low	High

### Model development

Of the 58 models identified, 49 were developed independently, while nine were derived from the existing models, of which five were derived from the NPI, one from Adjuvant!, one from IBTR! (the model predicts the risk of ipsilateral breast tumour recurrence), and two from PREDICT v1.1. The version PREDICT v1.2, also called PREDICT+, added HER2 status as a predictor into the first version PREDICT v1.1 [35]. The version PREDICT v1.3 added Ki67, a nuclear protein used as a marker of cell proliferation, into PREDICT v1.2 [36].

The models predicted mortality (*n* = 28), recurrence (*n* = 23), or both (*n* = 7), mostly based on participants in Europe (*n* = 25), followed by Asia (*n* = 13), North America (*n* = 12), and Australia (*n* = 1). Cox proportional hazards (PH) regression (*n* = 32) was the most commonly used method for model development, followed by artificial neural networks (*n* = 6), decision trees (*n* = 4), logistic regression (*n* = 3), and Bayesian methods (*n* = 3). The most commonly used predictors include nodal status (*n* = 49), tumour size (*n* = 42), tumour grade (*n* = 29), age at diagnosis (*n* = 24), and oestrogen receptor (ER) status (*n* = 21). The models were presented as regression formula (*n* = 13), followed by online tools (*n* = 8), decision trees (*n* = 5), nomograms (*n* = 4) and score chart (*n* = 1) (Table 4).

**Table 4:** Characteristics of the models

	Number of models^a^
Total	58 models
Types of models	
New models	49 models
Modified models	9 models
Year of development	1982–2016
1982–1989	5 models
1990–1999	11 models
2000–2009	17 models
2010–2016	25 models
Country of participants for model development	
Europe	25 models
Asia	13 models
North America	12 models
Others	1 model (Australia)
Unknown or from several trials	7 models
Method of model development	
Cox PH regression	32 models
Artificial neural networks	6 models
Decision tree	4 models
Logistic regression	3 models
Bayesian method	3 models
Multistate model	2 models
Support vector machine	2 models
Others	6 models
Outcomes	
Mortality	28 models
Recurrence	23 models
Both	7 models
Predictors	
Age at diagnosis	24 models
Nodal status	49 models
Tumour size	42 models
Tumour grade	29 models
Lympho-vascular invasion (LVI)	8 models
Stage	8 models
ER status	21 models
Progesterone receptor (PR) status	10 models
HER2 status	13 models
Treatment	17 models
Others	Mitotic activity index (MAI), histological subtypes, comorbidity, menopausal status, etc.
Presentation of model	
Regression formula	13 models
Online tool	8 models
Decision tree	5 models
Nomogram	4 models
Score chart	1 model
No report	27 models
Number of risk groups	
5	3 models
4	3 models
3	9 models
2	6 models
No report/No risk group	33 models
Validation	
No validation	11 models
Internal validation	43 models
External validation	17 models

^a^Total number of models is 58. Where each model can fit more than one category, the number of models may not always total 58

Seventeen models have been externally validated by independent researchers (*n* = 8) or by the model developers (*n* = 15). These models were developed to support clinical decision making (*n* = 14) or evaluating the prognostic value of specified clinical factors (*n* = 3) (Additional file 7). Additional file 8 presents the characteristics of these models.

The models that were most frequently validated include Adjuvant! (*n* = 17), the NPI (*n* = 15), and PREDICT v1.3 (*n* = 5). Among the 17 studies that externally validated Adjuvant!, three had high risk of bias in Prognostic Factor Measurements [35, 45, 46], one was at low risk of bias across the QUIPS domains [47], while the remaining studies had low or moderate risk of bias. Among the 15 studies that externally validated the NPI, three were at high risk of bias in Prognostic Factor Measurement [37, 48, 49], one was at high risk of bias in Statistical Analysis and Presentation [50], three were at low risk across the domains [47, 51, 52], and the rest had low or moderate risk of bias. All the five studies that externally validated PREDICT v1.3 had low or moderate risk of bias (Table 5).

**Table 5:** Risk of bias within the external validation studies by models

No	Model	Validated by	Authors(Year of publication)	Risk of bias domain			
				Study Participation	Prognostic Factor Measurement	Outcome Measurement	Statistical Analysis and Presentation
1	Adjuvant!	Model developer(s)	Mook et al. (2009) [64]	Low	Moderate	Moderate	Low
			Olivotto et al. (2005) [45]	Low	High	Low	Low
			Wishart et al. (2011) [46]	Low	High	Low	Low
			Wishart et al. (2012) [35]	Low	High	Low	Low
		Independent researcher(s)	Campbell et al. (2009) [59]	Low	Moderate	Low	Low
			Hajage et al. (2011) [58]	Moderate	Low	Moderate	Low
			Hearne et al. (2015) [47]	Low	Low	Low	Low
			M. Jung et al. (2013) [44]	Low	Moderate	Moderate	Low
			Laas et al. (2015) [68]	Moderate	Moderate	Moderate	Low
			Lende et al. (2010) [136]	Low	Moderate	Low	Low
			Plakhins et al. (2013) [63]	Moderate	Moderate	Low	Low
			Quintyne et al. (2013) [60]	Low	Moderate	Moderate	Low
			Rejali et al. (2015) [54]	Moderate	Moderate	Moderate	Low
			de Glas et al. (2014) [66]	Low	Moderate	Low	Low
			Bhoo-Pathy et al. (2012) [61]	Low	Moderate	Moderate	Low
			Kuo et al. (2012) [62]	Low	Low	Moderate	Low
			Mojir Sheibani et al. (2013) [65]	Low	Low	Moderate	Low
2	NPI	Model developer(s)	van Belle et al. (2010a) [73]	Low	Moderate	Low	Low
		Independent researcher(s)	Albergaria et al. (2011) [75]	Moderate	Moderate	Low	Low
			Balslev et al. (1994) [48]	Low	High	Low	Low
			Chollet et al. (2003) [49]	Moderate	High	Low	Low
			D’Eredita et al. (2001) [51]	Low	Low	Low	Low
			Hearne et al. (2015) [47]	Low	Low	Low	Low
			M. Liu et al. (2010) [74]	Low	Moderate	Low	Low
			Megha et al. (2010) [70]	Moderate	Moderate	Low	Low
			Okugawa et al. (2009) [52]	Low	Low	Low	Low
			Quintyne et al. (2013) [60]	Low	Moderate	Moderate	Low
			Rejali et al. (2015) [54]	Moderate	Moderate	Moderate	Low
			Sidoni et al. (2004) [71]	Moderate	Low	Low	Low
			Sundquist et al. (1999) [72]	Low	Moderate	Low	Low
			van Belle et al. (2010b) [37]	Low	High	Low	Low
			Yadav et al. (2015) [50]	Moderate	Moderate	Low	High
3	PREDICT v1.3	Model developer(s)	Wishart et al. (2014) [36]	Low	Moderate	Low	Low
		Independent researcher(s)	de Glas et al. (2016) [69]	Low	Moderate	Low	Low
			Laas et al. (2015) [68]	Moderate	Moderate	Moderate	Low
			Plakhins et al. (2013) [63]	Moderate	Moderate	Low	Low
			Wong et al. (2015) [67]	Low	Moderate	Low	Low
4	Cancer Math	Model developer(s)	Michaelson et al. (2011) [42]	Low	High	Low	Moderate
		Independent researcher(s)	Laas et al. (2015) [68]	Moderate	Moderate	Moderate	Low
			Miao et al. (2016) [138]	Moderate	High	Low	Low
5	MPI	Independent researcher(s)	Aaltomaa et al. (1983) [130]	Low	Low	Low	Low
			Carbone et al. (1999) [132]	Moderate	Low	Low	Low
			Collan et al. (1998) [98]	Moderate	Low	Low	Low
6	IBTR!2.0	Model developer(s)	Sanghani et al. (2010) [38]	Moderate	Moderate	Moderate	Low
		Independent researcher(s)	S.P. Jung et al. (2013) [134]	Low	Moderate	Low	Low
			Kindts et al. (2016) [135]	Moderate	Moderate	Moderate	Low
7	Paik et al. (1990)	Independent researcher(s)	Ribelles et al. (1997) [139]	Moderate	Low	Low	Low
8	Lovekin et al. (1991)	Independent researcher(s)	Ribelles et al. (1997) [139]	Moderate	Low	Low	Low
9	PREDICT v1.1	Model developer(s)	Wishart et al. (2010b) [40]	Low	High	Low	Low
			Wishart et al. (2011) [46]	Low	High	Low	Low
			Wishart et al. (2012) [35]	Low	High	Low	Low
10	PREDICT v1.2	Model developer(s)	Maishman et al. (2015) [137]	Moderate	Moderate	Low	Low
			Wishart et al. (2014) [36]	Low	Moderate	Low	Low
			Wishart et al. (2012) [35]	Low	High	Low	Low
11	iNPI	Model developer(s)	van Belle et al. (2010a) [73]	Low	Moderate	Low	Low
12	NPI+	Model developer(s)	Green et al. (2016) [133]	Low	Moderate	Low	Low
13	INFLUENCE	Model developer(s)	Witteveen et al. (2015) [39]	Low	Moderate	High	Low
14	OPTIONS	Model developer(s)	Campbell et al. (2010) [19]	Moderate	Low	Moderate	Moderate
15	Chen et al. (2016)	Model developer(s)	Chen et al. (2016) [41]	Low	High	Low	Low
16	de Laurentiis et al. (1999)	Model developer(s)	de Laurentiis et al. (1999) [43]	Moderate	Low	Moderate	Moderate
17	Bryan et al. (1986)	Model developer(s)	Alexander et al. (1987) [131]	Low	Low	Low	Low

Total number of validation studies is 49. Since some studies validated more than one model, the number of studies does not total 49

While the web-based programmes Adjuvant! and PREDICT v1.3 estimate the possible survival time for breast cancer patients, the NPI assigns a prognostic index (PI) score to each individual patient based on the calculation *(0.2x tumour size in cm) + lymph node stage + tumour grade*. Originally, the NPI was developed based on the lymph node stage, but later the authors suggested that the number of involved nodes can replace the lymph node stage [32]. At the outset, a patient will be classified into one of three prognostic groups based on their NPI score: good prognostic group (PI< 3.4), moderate prognostic group (3.4 ≤ PI≤5.4), and poor prognostic group (PI> 5.4) [53]. Some validation studies of the NPI further divided the samples into six smaller prognostic groups [47, 54].

### Model validation

#### Internal validation

Forty two models were internally validated by comparing the predicted outcomes to (a) the observed outcomes (*n* = 20); (b) the outcomes predicted by the NPI or Adjuvant! (*n* = 7); (c) the outcomes predicted by prognostic factors (*n* = 4); or (d) the outcomes predicted by other newly developed models (*n* = 15). The sampling methods for internal validation were cross-validation (*n* = 13), random-splitting (*n* = 11), or bootstrap (*n* = 5); some internal validation cohorts were exactly the same to the development cohorts (*n* = 13), or they were the development cohorts with longer follow-up (*n* = 1), or they were specific subgroups of the development cohorts (*n* = 1), or they were the combination of the development cohorts and the newly recruited patients in the same centres (*n* = 1), or they were different patients from the development cohorts but in the same hospitals (*n* = 1). The models were assessed for overall performance (*n* = 3), calibration (the level of agreement between the predicted and observed outcomes) (*n* = 12), discrimination (the extent to which a model can discriminate patients with the outcomes and those without the outcomes) (*n* = 28), and clinical usefulness (*n* = 13). Brier scores (*n* = 2), calibration plots (*n* = 7), Kaplan-Meier curves (*n* = 23), and accuracy rates (*n* = 11) were most commonly used to assess the models’ overall performance, calibration, discrimination, and clinical usefulness, respectively (Table 6**)**.

**Table 6:** Validation methods

Domain	Measure	Description	Internal validation	External validation
Overall performance		Measuring the distance between the predicted and actual outcomes [9]	3 studies	2 studies
	R^2^	The amount of variability in outcomes that is explained by the model [9]	1 study	1 study
	Brier score	A measure of the average discrepancy between the true disease status and the predicted probability of developing the disease [85]	2 studies	1 study
Calibration		The level of agreement between the observed and predicted outcomes [9]	12 studies	32 studies
	Calibration plot	Having predictions on the *x* axis, and the observed outcome on the *y* axis [9]	7 studies	20 studies
	SMR (Standardised mortality ratio)	The difference from the predicted calibration line and the ideal line in calibration plot [69]	0 study	1 study
	E/O	Ratio between the predicted and observed outcomes [100]	3 studies	2 studies
	E-O	Absolute difference between the predicted and observed outcomes	2 studies	28 studies
	Hosmer-Lemeshow goodness-of-fit test	The ability of a model to fit a given set of data [9]	4 studies	5 studies
Discrimination		The extent to which the model can discriminate patients with the outcome and those without the outcome [9]	28 studies	37 studies
	Kaplan-Meier curve	The probability of surviving in a given length of time while considering time in many small intervals [140]	23 studies	20 studies
	Log-rank test	Testing the null hypothesis that there is no difference between populations in the probability of an event at any time point [141]	16 studies	18 studies
	C-index	The probability that, for a randomly chosen pair of patients, the one who actually experienced the event of interest has a higher predicted value than the one who has not experienced the event [85]	11 studies	12 studies
	AUC	Area under the receiving operating characteristic curve is identical to C-index for a model with binary outcome [9]	11 studies	12 studies
	CPE	Concordance probability estimate represents the pairwise probability of lower patient risk given longer survival time [142]	0 study	1 study
Clinical usefulness		The ability to make better decisions with a model than without it [9]	13 studies	1 study
	Accuracy rate	$=\frac{true\ negative+ true\ positive}{Total\ patients}$ [9]	11 studies	1 study
	Sensitivity	The fraction of true-positive classifications among the total number of patients with the outcome [9]	9 studies	1 study
	Specificity	The fraction of true negative classifications among the total number of patients without the outcome [9]	8 studies	1 study
	Positive predictive value (PPV)	$=\frac{number\ of\ true\ positives}{number\ of\ positives\ calls}$	1 study	0 study
	Negative predictive value (NPV)	$=\frac{number\ of\ true\ negative s}{number\ of\ negative\ calls}$	1 study	0 study
Agreement		Measure the agreement when comparing two models	0 study	4 studies
	Kappa coefficient (κ)	Measuring the inter-rater agreement for qualitative items.	0 study	1 study
	Correlation coefficient (Pearson or Spearman)	Measuring how strong a pair of variables is related	0 study	3 studies
Others	Shrinkage factor	Cross-validated prognostic index [143]	2 studies	0 study
	Univariate analysis	Examining the distribution of cases in only one variable at a time	2 studies	10 studies
	Multivariate analysis	Examining more than two variables simultaneously	3 studies	6 studies

Overall, most models performed well in the internal validation cohorts, some even showed better performance than the existing models [19, 22, 37, 44, 55, 56] or prognostic factors [43, 53, 57].

#### External validation

Only 17 models have been externally validated by comparing the predicted outcomes with the observed outcomes (*n* = 35), with the outcomes predicted by other models (*n* = 10), or with the outcomes predicted by single prognostic factors (*n* = 4). Participants were recruited in countries different from the development cohorts (*n* = 39) or in the same countries but different centres/sources (*n* = 9). The models were assessed for overall performance (*n* = 2) (using explained variation R^2^ (*n* = 1) and Brier score (*n* = 1)); calibration (*n* = 32) (mainly using calibration plots (*n* = 20) and/or the comparison of the predicted (E) to the observed outcomes (O) (*n* = 30)); discrimination (*n* = 37) (mainly using Harrell’s C-index/AUC (Area under the Receiver operating characteristic (ROC) curve) (*n* = 22), Kaplan-Meier curve (*n* = 20), and/or log-rank test (*n* = 18)); and clinical usefulness (*n* = 2) (using accuracy rate (*n* = 2) and sensitivity/specificity (*n* = 1)). Some studies that compared two or more models tested the agreement between the models (*n* = 4), using Kappa coefficient (κ) (*n* = 1) and correlation coefficients (Pearson or Spearman) (*n* = 3). Univariate (*n* = 10) and multivariate analysis (*n* = 6) were used to test if prognostic factors and prognostic scores were significant to outcomes (Table 6). A summary of the external validation studies is presented in Additional file 9.

In general, the models performed less accurately in some independent populations, particularly in patients with high risk, in young and elderly patients. For example, Adjuvant! predicted prognosis accurately in patients from France [58], Canada [45, 46], and those with low grade tumours, but less accurate in patients from UK [59], Ireland [60], Malaysia [61], South Korea [44], Taiwan [62], those with lympho-vascular invasion [45, 61], BRCA1-mutation carriers [63], and those with high grade tumours [44, 58, 59, 61, 62]. Studies showed inconsistent results of Adjuvant! in patients aged 40 years or less [35, 44–47, 54, 58, 59, 61, 62, 64] and elderly patients [45, 46, 54, 59, 61, 65, 66]. Similarly, PREDICT v1.3 performed well in Malaysian patients [67], but less accurately in patients with BRCA1 mutations [63], patients aged 40 years or less [67], and those with ER positive and HER2 negative tumours [68], and inconsistently in elderly patients [67, 69]. An exception is the NPI, which performed well in most populations, including patients from Italy [51, 70, 71], Sweden [72], Denmark [48], Belgium [73], Norway [37], Japan [52], India [50], New Zealand [37], patients aged 40 years or less [47], metastatic patients [74], those with triple negative breast cancer [75], and those treated with neoadjuvant chemotherapy [49].

#### Studies that compared different models in independent datasets

In the three studies that compared the NPI and Adjuvant! conducted by independent researchers, no model was shown to be better than the other. One study showed that both models performed accurately in the overall cohort of Iranian patients, but less accurately in some subgroups [54]. Another study found that Adjuvant! showed better discrimination ability than the NPI in Irish breast cancer patients, although Adjuvant! underestimated the 10-year OS [60]. However, the third study showed that, in British breast cancer patients aged 40 years or less, the NPI’s prediction was nearly similar to the observed outcomes, while Adjuvant! seemed to overestimate the 10-year OS, although the study power was not sufficient to generate a statistically significant difference [47] (see details in Additional file 10).

None of the three models compared by independent researchers– PREDICT v1.3, Adjuvant!, and CancerMath– was found to be superior. In the studies that compared PREDICT v1.3 and Adjuvant!, both did not predict the 10-year OS well in BRCA1-mutation carriers [63] and in patients aged 65 years or more [66, 69], with statistically significant differences between the predicted and observed outcomes (*P* < 0.05). PREDICT v1.3 accurately predicted the 5-year OS in elderly patients, though not in all subgroups, but the authors could not compare that model with Adjuvant! because the latter did not predict the 5-year OS [69]. When PREDICT v1.3, Adjuvant!, and CancerMath were compared in patients with ER positive and HER2 negative tumours, all the three models inaccurately predicted the 10-year OS, with statistically significant differences between the predicted and observed outcomes (*P* < 0.05) [68] (see details in Additional file 10**)**.

There are four studies that developed new models, and then compared them to existing models in independent datasets (see details in Additional file 11). In its development study, PREDICT v1.1 showed better performance than Adjuvant! in predicting 10-year breast cancer specific survival (BCSS), but poorer performance in 10-year OS in the overall cohort [46]. PREDICT v1.1 was better in some sub-groups (10-year OS in patients with grade 3 tumours, lymphovascular positive tumours, and node negative tumours; 10-year BCSS in patients with node positive tumours, tumour size > 21 mm, and ER positive tumours), whereas Adjuvant! was better in others (10-year OS in patients with tumour size > 21 mm, grade 2 tumours, and ER positive tumours; 10-year BCSS in patients with grade 3 tumours, ER negative tumours, and node negative tumours) [46]. In its development study, PREDICT v1.2 showed significantly better performance than PREDICT v1.1 and Adjuvant! in the HER2 positive subgroup, possibly because it was developed by adding HER2 status as a prognostic factor into PREDICT v1.1 [35]. However, in the overall cohort, Adjuvant! was better in predicting OS while both versions of PREDICT were better in predicting BCSS [35]. The development study of the iNPI showed that this version discriminated slightly better than the original version NPI, but the difference was not significant [37]. The development study of PREDICT v1.3 showed that this new version improved both calibration and discrimination compared to the previous version PREDICT v1.2 in patients with ER positive tumours [36].

## Discussion

This study reviewed 96 articles that presented the development and/or validation of prognostic models for breast cancer. To our knowledge, this is the most comprehensive review of prognostic models for breast cancer. A previous review reported only six models based on clinico-pathological factors [14]. However, our findings may be affected by publication bias [8, 76] as well as the diversity of terms used in prognostic research [14, 77]. The review may have missed some relevant studies that were published after December 2016, for example, PREDICT v2.0, which added age at diagnosis as a predictor into PREDICT v1.3 [78].

Due to the heterogeneity of study designs, inclusion criteria, measurement techniques, methods of analysis, and methods of handling of continuous variables, meta-analysis was not undertaken as recommended previously [76, 79]. Instead, we assessed the risk of bias for each individual study using the modified QUIPS tool.

The original QUIPS tool was developed to assess bias in studies establishing the relationship between a prognostic factor and an outcome [17], in which confounders may play an important role. In contrast, we are interested in outcome prediction studies where causality and confounding are not a concern [9]. Therefore, we did not assess the confounding issue of the selected articles. We also omitted the domain of Study Attrition because, although most of the selected studies described attempts to track loss to follow-up to some extent, none of them reported specific information required by the QUIPS tool (including: the proportion of study sample dropping out of the study, attempts to collect their information, reasons for loss to follow-up, their key characteristics, and if these characteristics are different from those who completed the study [17]).

We found that most studies were at moderate or low risk of bias, which contrasts with the findings in other systematic reviews that most studies were at poor quality [11, 77]. However, the previous reviews did not report the detailed quality assessment of each study.

Most studies included in this review used a retrospective design, and therefore had issues related to missing data and a lack of consistency in predictor and outcome measurement [9, 11, 77]. Prospective cohort studies have been suggested as the best design for predictive modelling because they enable not only clear and consistent definitions but also prospective measurement of predictors and outcomes [3, 9].

Similar to the previous systematic reviews [8, 77, 80, 81], we found that most studies (59%) did not report, or did not satisfy the suggested minimum requirements for the numbers of events, i.e., 10 events per candidate variable for model development studies, and 100 events for model validation studies [11, 82–87]. A small number of events could mislead the results of validation measures, for example, misleadingly high value of the C-index [85].

We found that the most commonly used prognostic factors in the models were nodal status, tumour size, and tumour grade, followed by age at diagnosis and ER status, as reported in other reviews [11, 88]. The NPI was one of the simplest and oldest models, and included only nodal status, tumour size, and tumour grade. There are several attempts to improve the prognostic values of the NPI by adding other novel predictors, such as age at diagnosis [89], hormonal receptor status [37, 89, 90], and HER2 status [37, 55, 90, 91]. However, such modification has not been proven to be better than the NPI in independent populations. Future research may evaluate the added prognostic value of other important variables to the NPI and other models.

The use of gene expression or novel biomolecular factors is increasing due to their potential to provide molecular phenotyping that recognises distinct tumour categorisations not evident by traditional factors [92, 93]. However, we excluded models based on genetic profiles or novel biomolecular factors because these factors are not yet widely adopted in clinical practice. Additionally, since models that include both genetic and traditional factors are suggested to be superior to those based on either set of features alone [94, 95], studies of the prognostic value of any new marker should look at the extra benefit of including it when traditional clinico-pathological variables are also included.

The most commonly used method for model development was Cox PH regressions as reported in other reviews [11, 96]. Cox PH regressions are simple but have been criticised because the PH assumption may not always hold, since the strengths of prognostic factors change over time in the “real world” [19, 29, 97]. To address this, alternative methods such as artificial neural networks, support vector machines, or multistate models have been applied. These models may perform better than Cox PH models but have not been validated in independent populations, limiting generalisability [22–24, 26]. Furthermore, clinical validity is more important than statistical validity [11]. As the models developed based on Cox PH regressions, such as the NPI or PREDICT, showed good performance in many populations, Cox PH regressions will still dominate the literature on model development methods.

Differences in the methodological issues pointed out in our review may be explained by differences in the purpose of developing the model (e.g., to support clinical decision making, to evaluate the prognostic value of a specific factor, or to compare statistical methods used to develop the model). However, not many developers explicitly stated the purposes of their models. Nevertheless, the models that have gone to further external validation were developed mainly to support clinical decision making. These models were considered useful in clinical practice.

Only one of 49 external validation studies in our review tested “clinical usefulness”, which was defined by the authors as the ability for a model to classify patients into low risk and high risk groups better than without that model, and the measure used was accuracy rate [98]. However, a model’s ability to classify patients into two risk groups may not reflect its usefulness in clinical settings. A prognostic model can be useful if it classifies patients into more than two risk groups to influence therapy or to save patients from unnecessary treatments or to estimate survival time for patients [8]. Future research may consider more relevant measures to assess clinical usefulness such as the improvement of clinical decision making when applying a model, patients’ insights about model reports, or how doctors communicate with patients about model results.

Previous reviews reported that Hosmer-Lemeshow goodness-of-fit test was used most frequently to test the deviations in calibration plots [77, 81] but we found that the difference between the predicted and observed outcomes was more commonly used (Table 6). Steyerberg and Vergouwe (2014) did not recommend the Hosmer-Lemeshow goodness-of-fit test because it only provides a *p*-value instead of providing the direction and magnitude of miscalibration [99]. This test has also been criticised for being arbitrary and imprecise as the p-value is dependent on miscalibration and sample size [99]. Instead, Steyerberg and Vergouwe (2014) advocated the use of the intercept of the calibration plot, also called calibration-in-the-large [99], which is closely related to the difference between the predicted and observed outcomes, either absolute or relative difference [100].

We found that C-index/AUC was the most commonly used method to assess discrimination, followed by Kaplan-Meier curves and log-rank tests, as reported in previous systematic reviews in several clinical fields [9, 77, 96]. Log-rank tests were not recommended because they do not give an estimate of the magnitude of the separation of the risk groups [96]. In contrast, C-index, or AUC for a binary endpoint, was advocated by several authors [99].

This review focused on models that have been externally validated in several settings by independent researchers for many reasons. Firstly, external validation is preferable to internal validation to test a model’s transportability as the case-mix (or the distribution of predictors) in an independent population is unlikely to be identical with that in the model development population [85]. Secondly, to enhance the generalisability of a model, it should ideally be validated in different settings with diversity of case-mixes [85]. A model with good performance in diverse settings is more likely to be generalisable to a plausibly related, but untested population [13, 85, 86]. Finally, a reliable model should be tested by independent researchers in different settings [8, 101]. If model development and external validation are undertaken by the same researchers, there may be a temptation to revise the model to fit the external validation data [8]. A clear distinction between the external validation studies conducted by independent researchers and by model developers should be made to reduce inflated findings and “spin” [102–104].

The studies that compared Adjuvant!, CancerMath, PREDICT v1.3, and the NPI in independent datasets by independent researchers did not find the superiority of one model over the others. When they were validated individually, only the NPI performed well in most independent populations, whereas the other models were accurate in just some populations. The NPI has been advocated by several authors and is one of the few models that are used in clinical practice [11]. The advantage of the NPI is its simplicity, which is an important criterion in developing a useful model [105]. Additionally, the model shows good reducibility and transportability because it performed well in diverse settings when validated by independent researchers. The model has good discrimination in most populations, and is therefore clinically useful because it classifies patients into risk groups to influence therapy or save patients from unnecessary treatments [8, 11]. However, most studies that validated the NPI only assessed its discrimination but not calibration, because the model cannot estimate prognosis of individual patients. Some studies assigned OS for all patients in the same NPI group based on previous reports [47, 54, 73]. This practice is criticised as inappropriate, since estimates based on data at a period in the past are probably not well calibrated for patients today. Advanced treatments, such as hormonal therapies or targeted therapies, in addition to improvement in detection and diagnosis, may improve the survival within the NPI groups [106]. Regular updates would be required for better prediction of prognosis for each group.

The performance of a particular model may vary across different populations. For example, the NPI, a UK-based model, performed well in most countries in Europe (Italy, Sweden, Denmark, Belgium, Norway), and even in Asia (Japan, India), but was less accurate in Irish patients. The US-based model Adjuvant! showed good performance in a large Dutch population, but poor performance in patients from the UK or Asia (Malaysia, South Korea, Taiwan). Therefore, a reliable validation study should be conducted before a model is applied in other populations.

Most studies in our review showed that models were less accurate in patients aged under 40 years or over 65 years, although some studies showed opposite results. Likewise, a previous review concluded that Adjuvant! was less accurate in young and elderly patients in most studies [14]. However, most validation studies lack generalisability because they were based on small numbers of events or did not report the numbers of events. Only a few studies with appropriate numbers of events were designed to assess models’ performance in young and elderly patients only. These studies found that PREDICT v1.3 was less accurate in predicting 10-year OS [69], whereas Adjuvant! overpredicted 10-year OS and event-free survival (EFS) in Dutch elderly patients [66]. Nonetheless, it is difficult to know if the poor performance of models in young and elderly patients was attributable to age only, or to other effect modifiers such as ethnicity.

## Conclusion

We reviewed the development and/or validation of 58 models predicting mortality and/or recurrence for female breast cancer. These models varied in terms of methods of development and/or validation, predictors, outcomes, and patients included. Most models have been developed in Europe, Asia, and North America. We found that models performed well in internal validation cohorts, but the results were unpredictable in external validation cohorts, especially in young and elderly patients, and in high risk patients. NPI is an exception, which performed well in most independent populations. Therefore, models should be validated before being applied in another population.

## Additional files

Additional file 1: Search terms. (XLSX 10 kb)

Additional file 2: Model development studies that were excluded because of no full text. (XLSX 13 kb)

Additional file 3: Model validation studies that were excluded because of no full text. (XLSX 12 kb)

Additional file 4: Articles that presented model development and/or internal validation. (XLSX 47 kb)

Additional file 5: Articles that presented only the internal validation. (XLSX 12 kb)

Additional file 6: Articles that presented model external validation. (XLSX 39 kb)

Additional file 7: Number of external validation studies. (XLSX 10 kb)

Additional file 8: Characteristics of the models validated in external populations. (XLSX 11 kb)

Additional file 9: Overview of external validation studies. (XLSX 16 kb)

Additional file 10: Studies that compared models by independent researchers. (XLSX 10 kb)

Additional file 11: Studies that compared models by models’ developers. (XLSX 9 kb)

## Abbreviations

Adjuvant!: Adjuvant!Online
AIC: Akaike information criterion
AUC: Area under the curve
BCSS: Breast cancer-specific survival
BIC: Bayesian information criterion
Cox PH regression: Cox proportional hazards regression
CPE: Concordance probability estimate
DFS: Disease-free survival
EFS: Event-free survival
ER: Oestrogen receptor
HER2: Human epidermal growth factor receptor 2
IBTR: Ipsilateral breast tumour recurrence
LRR: Local-regional recurrence
MAI: Mitotic Activity Index
MPI: Morphometric Prognostic Index
NPI: Nottingham Prognostic Index
NPV: Negative predictive value
OS: Overall survival
PPV: Positive predictive value
PR: Progesterone receptor
QUIPS: QUality In Prognosis Studies
RCT: Randomised controlled trial
RFS: Relapse-free survival
ROC: Receiver operating characteristic
SD: Standard deviation
SE: Standard error
SMR: Standardised mortality ratio
The UK: The United Kingdom
The USA: The United States of America
TNM: Tumour, Node, and Metastasis
